# Welfare in Swiss dairy heifers: Comparative assessment of skin lesions in 2 housing systems

**DOI:** 10.3168/jdsc.2024-0610

**Published:** 2024-08-16

**Authors:** R.M. Schmid, A. Loup, E. Studer, J. Becker

**Affiliations:** Clinic for Ruminants, Vetsuisse Faculty, University of Bern, 3012 Bern, Switzerland

## Abstract

During summer, dairy heifers are often managed extensively in Switzerland. This applies to 2 different types of husbandry systems across the country (i.e., to heifers on alpine transhumance pastures and to heifers on pastures belonging to a year-round lowland farm). In contrast to heifers, dairy cows are more intensively farmed and spend more time in confinement. The prevalence of skin lesions (e.g., hock injuries) in dairy cows is high. The aim of this study was to record the prevalence of skin lesions in heifers and to possibly determine the time of occurrence of these lesions. To this end, heifers from both husbandry systems (alpine pastures vs. year-round lowland farm pastures) were inspected both visually and through palpation, and findings were compared. Inspection took place in the fall (i.e., at the end of the alpine pasturing season before descent to the lowlands). Of the 480 heifers included on a total of 28 farms, skin lesions were found in 13.1% of individuals. Skin lesions commonly associated with barn design (e.g., injuries on the dewlap or hock injuries) and skin lesions on the pelvis were most frequently detected, equally distributed among husbandry systems. In conclusion, the prevalence of skin lesions among Swiss dairy heifers appears to be lower compared with dairy cows, at least as measured in the fall before heifers transition to the main dairy herd. Accordingly, deploying measures to reduce skin lesions is more efficient on dairy farms than in heifer raising systems.

## Highlights

• Heifers have few skin lesions in mountain and lowland farms.
• Skin lesions in dairy cows should be addressed on dairy farms.
• Heifers can handle harsh mountain terrain well.
• Alpine pasturing is not associated with lower welfare.

## Graphical Abstract

**Figure:**
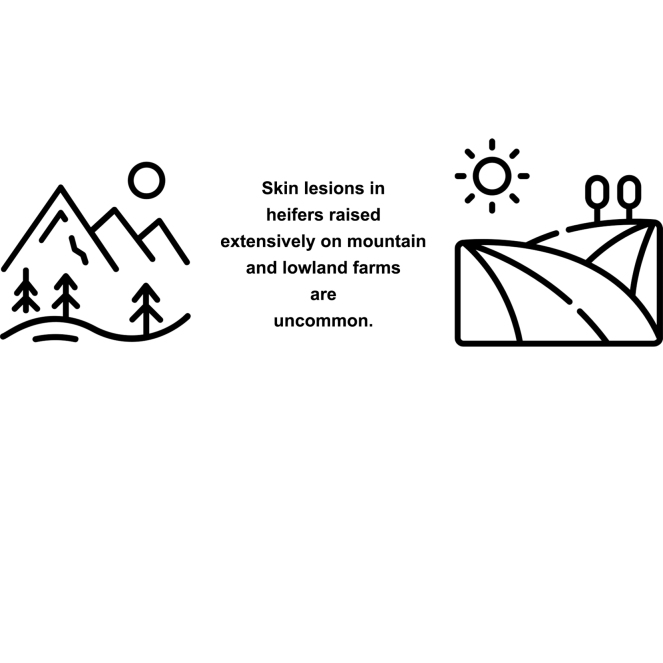
**Summary:** In Switzerland, heifers are frequently raised in extensive systems. During transhumance, many spend summer months in remote high-alpine pastures, where topographic structure and weather conditions may be harsh. Others remain in lowland farms all year. We investigated the prevalence of skin lesions (chronic and acute injuries) in heifers in both mountain and lowland settings. Overall prevalence was low (13.1%), with most lesions found at dewlap and tarsal joints. No difference between alpine and lowland farms was observed. In contrast to the prevalence of skin lesions in dairy cows, skin lesions in heifers are rare, which indicates that measures to reduce them should be deployed in dairy farms for the highest efficiency, rather than on farms raising heifers.

Each year, in early summer, around 120,000 Swiss dairy heifers are sent to alpine transhumance pastures, where they traditionally spend 100 d until fall (Federal Office for Agriculture, 2024). There, they usually have a large grazing area at their disposal, in addition to occasionally available barns in case of adverse weather conditions. It is not yet known whether the challenging topography and terrain conditions of mountain pastures or the barn equipment on alpine pastures are associated with increased skin lesions. Generally, studies on skin lesions in heifers on both alpine pastures and on pastures belonging to a lowland farm are scarce. On the latter, heifers often spend the summer on pastures in easier terrain. Similar to findings of another study, the presumably much greater space allowance on alpine pastures could be associated with a lower prevalence of skin lesions (Boyle et al., 2008). The aims of the present study were (1) to compare the prevalence of skin lesions between Swiss heifers kept on alpine pastures and lowland pastures in the end of the alpine pasture season before the descent to the valley, and (2) investigate risk factors for the occurrence of skin lesions in Swiss heifers during summer. The present study was conducted in the frame of an epidemiological investigation of indicator bacteria exhibiting antimicrobial resistance, of which the results will be published elsewhere (animal experiment approval BE128/2022). Participation was called for in the national agricultural press and by the Swiss Alpine Association. Participation was nonrandomized, voluntary, and not remunerated. Farms were included between May 2023 and July 2023, and data were collected between August 2023 and October 2023. Enrolled heifers were inspected and palpated for skin lesions. These could possibly be associated with contact with stable equipment including lying surfaces (typical locations of the body, e.g., tarsus, knee, spine, carpus, withers, dewlap) or with contact with sharp-edged items or falls on steep terrain (Clavadetscher et al., 2023).

A total of 14 alpine farms and 14 lowland farms in the cantons of Argovia, Appenzell Inner Rhodes, Basel, Bern, Fribourg, Lucerne, Schwyz, Thurgovia, and Valais were included. Alpine farms were visited, and heifers were examined within 7 d before the end of transhumance. Heifers on the lowland pastures were examined at the same time of year. Only weaned female bovines not having given birth (heifers) were included. Heifers were intended for continued dairy production. Pregnancy status was not evaluated. Detailed characteristics of the husbandry system were assessed by inspection and by means of a questionnaire, which was completed with the farm manager. The questionnaire was carried out in the native language of the farm manager and had been validated in advance by experts. It included questions concerning the type of barns available, the surface quality and bedding of the lying areas, the frequency of pasture access, and the pasture size, among others.

On small-scale farms (herd size ≤20 heifers), all heifers were examined. On medium-scale farms (herd size >20 to herd size ≤40), 20 randomly selected heifers were included, whereas on large-scale farms (herd size >40 heifers), 50% of the heifers were randomly selected. Random selection was carried out by sorting the respective list of heifers' ear tag numbers using Microsoft Excel (Microsoft, Redmond, WA) and choosing the required number of heifers starting from the top. Included heifers were inspected and palpated for skin lesions. This procedure was applied to examine at least 50% of the heifers on each farm with a reasonable cost-benefit ratio. The maximum number of inspected heifers per farm was 30. Heifers in both husbandry systems were examined once after 103.2 ± 23.8 d on pasture (alpine farms) and 107.4 ± 25.8 d on pasture (lowland farms; mean ± SD; see Table 1). Data collection was performed according to a standardized scheme (Cows and More, https://cowsandmore.com/). We assume that skin injuries which had occurred before the pasture period would have healed by the time of data collection in fall. Observed skin lesions would therefore either be attributed to the respective present husbandry system (Livesey et al., 2002) or the topography of the pasture (Clavadetscher et al., 2023).

**Table 1:** Farm characteristics of 28 farms raising heifers in Switzerland enrolled in an investigation of skin lesions during the summer grazing period, 2023^1^

Parameter	No. of farms		Location	Mean	SD	Median	Min	Max
Enrolled animals	14		Alpine	15	4.6	15	8	20
	14		Lowland	19.3	5.4	20	8	30
	28		Total	17.1	5.3	18.5	8	30
Age (mo)	14		Alpine	21.5	6.1	21.6	8.4	35.8
	14		Lowland	15.8	5.7	15.4	4.4	34.2
	28		Total	18.3	6.5	18.6	4.4	35.8
Mean pasture area (ha per heifer)	14		Alpine	21.9	18.7	14	8	70
	14		Lowland	6.6	7.4	4	0	28
	28		Total	14.2	16.0	9.8	0	70
Mean time on pasture (d)	14		Alpine	103.2	23.8	104.0	65.0	139.0
	14		Lowland	107.4	25.8	104.5	63.0	155.0
	28		Total	105.3	24.5	104.5	63.0	155.0
Percentage of Holstein breed	14		Alpine	28.7	36.4	20	0	95
	14		Lowland	59	37.1	67.5	6.7	100
	28		Total	43.9	39.2	24.6	0	100

1 Min = minimum; Max = maximum.

Descriptive statistics were performed using the chi-squared test with Yates continuity correction for categorical data and Mann–Whitney U test for ordinal scaled data at a significance level of 5% (Social Science Statistics calculator, available at https://www.socscistatistics.com/).

Analytical statistics were performed to model the presence or absence of skin lesions in a binomial (binary) model with link function (glmer) in ‘R' using the lme4 package (R Core Team, Vienna, Austria). The potential predictor variables were offered to the model at the animal level (age, breed) and at the farm level (type of barn, type of stall, main abode [pasture or barn], permanent access to pasture (yes or no), permanent access to barn (yes or no), and “observer” to check for observer bias). A random effect for heifer nested in farm was included.

A total of 480 heifers were included in the study (210 on alpine pastures and 270 on lowland pastures, i.e., 43.8% and 56.3%, respectively). Of these, 27% were Holstein-Friesian, 21% Red Holstein, 21% Brown Swiss, 11% Simmental, 10% Swiss Fleckvieh, and 10% belonged to a total of 7 other breeds or crossbreeds of the aforementioned. On average, heifers were provided an area of 1.42 ± 1.33 ha on alpine pastures compared with 0.30 ± 0.50 ha for heifers on lowland pastures (mean ± SD).

At the time of examination, heifers were aged 18.29 ± 6.52 mo (mean ± SD; minimum: 4.43, maximum: 35.77). On alpine farms, 131 heifers (62.4%) were temporarily kept in tiestalls in case of adverse weather conditions (e.g., thunderstorms, midday heat on hot days), whereas 44 heifers did not have access to a barn (21.0%) and 35 had access to freestall barns (16.7%). All 270 heifers on lowland farms had access to freestall barns (100%). Permanent access to pasture was provided on all alpine farms, and for 227 heifers on lowland farms (84.1%). There was no permanent access to pasture for 43 (15.9%) heifers on lowland farms. Of all heifers on alpine farms, 71.9% were mainly kept on pasture, whereas no precise data were available for 28.1% of heifers. On lowland farms, the pasturing management and the available structures differed considerably on farm level (25.2% mainly on pasture, 74.8% mixed systems with barn access). For this reason, the data are presented at animal level.

On alpine farms, 76 (36.2%) heifers were provided wooden floor without straw bedding and 9.5% wooden floor with straw bedding. A rubber mat with straw topping was provided to 9.5% of heifers, and without straw topping to 16.7% of the heifers. A special softer rubber mat approved as equivalent to straw bedding by the competent authority (so-called “BTS” mat) with lime topping was available to 7.1% of heifers on these farms (The Swiss Confederation, 2020).

On lowland farms, lying areas or cubicles were offered to 242 of the heifers (89.6%) with various bedding materials (straw, lime straw, dried manure solids, and sawdust) and stalls with rubber mats were used (with and without straw bedding; for 7.4% and 3.0% of heifers, respectively).

The prevalence of heifers with at least one skin lesion was 13.1% (n = 63). Of the heifers showing skin lesions, 34 were kept on alpine farms and 29 on lowland farms, whereby no statistically significant difference was found between the 2 husbandry systems χ^2^ = 2.62 (df = 1, number of observations [N] = 480), *P* = 0.11. Affected heifers suffered from an average of 1.36 ± 0.81 skin lesions (alpine farms: 1.52 ± 0.99; lowland farms 1.17 ± 0.47), although no significant difference was found between the husbandry systems (value of U = 414, z-score = 1.04, *P* = 0.30). Observed skin lesions are listed in Table 2. The most common findings were lesions on the dewlap, followed by lesions at the tarsus and carpus. Also, skin lesions elsewhere on front and hind limbs, pelvis, and head have been recorded. Furthermore, several other findings included papilloma (20), dermatophytosis (8), horn lesions (capital horn; 2), and swollen limbs of unknown etiology (2) were recorded. These secondary findings did not contribute to the calculation of the prevalence of skin lesions.

**Table 2:** Body locations and prevalence of skin lesions in 480 Swiss heifers on alpine and lowland farms, 2023^1^

Location of skin lesions	Alpine farms (n = 210)	Lowland farms (n = 270)	Total (n = 480)
Dewlap	12 (5.7%)	7 (2.6%)	19 (4%)
	(Min: 0; Max: 1)	(Min: 0; Max: 1)	
Carpi	7 (3.3%)	6 (2.2%)	13 (2.7%)
	(Min: 0; Max: 2)	(Min: 0; Max: 2)	
Front limbs other than carpi	4 (1.9%)	1 (0.4%)	5 (1%)
	(Min: 0; Max: 2)	(Min: 0; Max: 1)	
Neck	0 (0%)	1 (0.4%)	1 (0.2%)
	(Min: 0; Max: 0)	(Min: 0; Max: 1)	
Withers	0 (0%)	2 (0.7%)	2 (0.4%)
	(Min: 0; Max: 0)	(Min: 0; Max: 1)	
Head	0 (0%)	3 (1.1%)	3 (0.6%)
	(Min: 0; Max: 0)	(Min: 0; Max: 2)	
Tarsi	9 (4.3%)	5 (1.9%)	14 (2.9%)
	(Min: 0; Max: 2)	(Min: 0; Max: 1)	
Knee	4 (1.9%)	2 (0.7%)	6 (1.3%)
	(Min: 0; Max: 2)	(Min: 0; Max: 1)	
Hind limbs other than tarsi/knees	7 (3.3%)	1 (0.4%)	8 (1.7%)
	(Min: 0; Max: 1)	(Min: 0; Max: 1)	
Croup	3 (1.4%)	6 (2.2%)	9 (1.9%)
	(Min: 0; Max: 3)	(Min: 0; Max: 1)	
Tail	6 (2.9%)	0 (0%)	6 (1.3%)
	(Min: 0; Max: 2)	(Min: 0; Max: 0)	

1 Min = minimum; Max = maximum.

As for analytical analysis of risk factors for skin lesions, no potential predictor variable was significantly associated with the outcome in a univariable analysis at a level of significance of 5%. Therefore, no model has been built and, consequently, we cannot report on key risk factors for skin lesions in heifers.

Reports on skin lesions of heifers in Switzerland and abroad are rare. Therefore, we use data from assessments of skin lesions in dairy cows for comparison purposes. These assessments often contain information on limb and skin health (hairless areas and skin lesions, respectively) and partly show significantly higher prevalence for Swiss dairy cattle (8.6%–100% and 0%–34.4%, respectively; Brenninkmeyer et al., 2013; Bernhard et al., 2020; Nuss et al., 2020), as well as in dairy cattle in other countries of Central Europe (35.6%–68% and 22%–68%, respectively; Kester et al., 2014; Ekman et al., 2018). Also, higher prevalence has been reported in countries outside of Europe (Weary and Taszkun, 2000; Fulwider et al., 2007; Kester et al., 2014). For dairy cows in mountainous areas of the Eastern Alps and dairy farms with outdoor grazing, there are prevalence rates that correspond to those of the heifers in this study (9%, 31%; and 3%, 17%, respectively) (Popescu et al., 2013; Spigarelli et al., 2021). In addition, increased outdoor exercise or grazing appears to be associated with a lower prevalence of skin lesions (Spycher et al., 2002; Regula et al., 2004; Burow et al., 2013; Bernhard et al., 2020). This also applies to farms that provide their cows with sufficient bedding (Regula et al., 2004; Barrientos et al., 2013; Brenninkmeyer et al., 2013).

Given a low prevalence and a low number of skin lesions per heifer, we assumed that heifers undergo only a limited amount of pain and suffering due to skin lesions. Most frequently observed skin lesions, localized on the dewlap, tarsus, and carpus may have been caused primarily by lying on hard ground and could be reduced by using softer surfaces, deeper bedding, or both (Livesey et al., 2002). Whether skin lesions or injuries (or both) that develop during the summer heal fast and spontaneously could not be answered using this study design.

The low prevalence may be due to frequent grazing, and the large pasture area is in line with another study in freshly calved heifers (Livesey et al., 2002). It should be noted that the present data were collected in the fall. In contrast to the heifers described by Boyle et al. (2008), most heifers in Switzerland frequently come into contact with barn buildings and equipment, especially during winter, because the temperate climate does not allow for year-round outdoor husbandry. This could possibly evoke skin lesions. However, studies on Swiss heifers during the winter months are lacking. The extensive data on skin lesions in dairy cows provide evidence that the prevalence of such alterations increases with the time spent indoors. The present study shows that Swiss heifers rarely transition with predamaged skin, at least in the fall, and that measures to prevent skin lesions directly deployed in dairy cow housing systems probably have the greater benefit.
